# Knockdown of Circular RNA hsa_circ_PVT1 Inhibited Laryngeal Cancer Progression via Preventing wnt4/β-Catenin Signaling Pathway Activation

**DOI:** 10.3389/fcell.2021.658115

**Published:** 2021-07-16

**Authors:** Feng Yu, Ying Lin, Mao-Mao Ai, Guo-Jie Tan, Jia-Li Huang, Zi-Rou Zou

**Affiliations:** ^1^Department of Otolaryngology Head and Neck, Guangzhou Red Cross Hospital, Jinan University, Guangzhou, China; ^2^Department of Otolaryngology Head and Neck, Guangzhou Twelfth People’s Hospital, Guangzhou Medical University, Guangzhou, China; ^3^Department of Otolaryngology Head and Neck, Shenzhen Longhua District Central Hospital, Guangdong Medical University, Shenzhen, China

**Keywords:** has_circ_PVT1, laryngeal cancer, miR-21-5p/CBX4, wnt4/β-catenin signal pathway, cell proliferation

## Abstract

**Aim:**

To explore the function and mechanism of circular has_circ_PVT1 on laryngeal cancer (LC).

**Methods:**

Microarray chip was performed to screen the differential expression of circRNA. Western blot and qRT-PCR was employed to detect the protein and mRNA level. CCK-8, clone formation, cell cycle, wound healing, and Transwell assay were performed to detect the cell proliferation, migration, and invasion ability. Luciferase assay and Fish were used to confirm the relationship between circ_PVT1/CBX4 and miR-21-5p. Flow cytometry and TUNEL assay were carried out to assess the apoptosis level.

**Results:**

The upregulation of circ_PVT1 was found in LC tissues and cells. Silencing of circ_PVT1 inhibited LC progression via targeting miR-21-5p and indirectly controlling CBX4. Wnt4/β-catenin signal pathway was inactivated by inhibiting the expression of circ_PVT1.

**Conclusion:**

Knockdown of circ_PVT1 prevented LC progression via targeting miR-21-5p/CBX4 by inhibiting wnt4/β-catenin signal pathway, which could provide a novel therapeutic target for LC.

## Introduction

Laryngeal cancer (LC) is one of the most common malignant tumors of the head and neck, accounting for 50% of head and neck tumors; squamous cell carcinoma (LSCC) is the most common, accounting for about 95% (Lefebvre, 2006; Steuer et al., 2017). With a high smoking rate, environmental pollution, an aging population, and the acceleration of industrialization, the rates of LC is becoming more and more serious, and the morbidity and death of LC is not optimistic (Carew and Shah, 1998). Late patients may have difficulty in pronunciation, breathing, and swallowing, and may be treated with partial or total laryngectomy, tracheotomy, or radiotherapy, which bring great psychological and physical distress to the patients, thus reducing their quality of life (Mendenhall et al., 1990). In the past 40 years, the 5 year survival rate has shown a downward trend. Tumor invasion and metastasis is an important cause of death in cancer patients, so the search for specific and effective tumor markers of LC has become one of the hotspots of LC research.

Circular RNA (circRNA) is a newly discovered endogenous non-coding RNA. In animals, circRNA is a rich, stable, and ubiquitous non-coding RNA that connects the 3′ and 5′ ends to form a complete covalent ring structure through the cyclization of exons or introns (Guarnerio et al., 2019; Kristensen et al., 2019; Vo et al., 2019). With the development of ribonucleic acid deep sequencing and bioinformatics, it has been found that circRNA is not an accidental mismatch in the process of transcription. It is expressed in a variety of organisms and plays different biological roles, especially in the formation and deterioration of tumors (Jeck and Sharpless, 2014). At present, it has been found that the main functions of circRNA are: (1) to act as an endogenous miRNA “sponge,” (2) to participate in transcriptional regulation, and (3) to participate in protein translation. Whether circRNA has other functions remains to be studied. The research on the relationship between circRNA and tumor is mainly focused on the function of circRNA acting as an endogenous miRNA “sponge.” CircRNA contains miRNA binding sites, which can be competitively combined with the same miRNA, through miR binding NA response element (microRNA response element, MRE), to relieve or reduce the inhibition of miRNA on target genes and regulate the expression of target genes (Beermann et al., 2016). This mechanism is called competitive endogenous RNA (ceRNA) hypothesis. “Sponge” ceRNA all contain specific and complementary binding sites to MRE, belonging to linear RNA molecules. Because the binding sites of “sponges” are specific to miRNA binding regions, these RNA molecules express these binding regions, thus adsorbing specific types of miRNA like sponges (Conn et al., 2015). Therefore, circRNA can act as a competitive inhibitor (similar to ceRNA) to inhibit miRNA, and thus affect the target miRNA downstream of miRNA to produce biological effects.

At present, there are few studies on the relationship between circRNA and LSCC. CircRNA has been proven to have the function of being a miRNA molecule “sponge” and being an antisense transcript of cerebellar degeneration associated protein 1 antisense transcript (antisense to the cerebellar degeneration-related protein 1 transcript, CDR1as, and antisense transcript of sex determination region Y (sex-determining region Y). CircRNA ciRS-7 (also known as CDR1as) is the first circRNA, with biological effects, which contains multiple tandem miR-7 binding sites, so it can be used as an endogenous miRNA sponge to inhibit miR-7 activity and participate in gene post-transcriptional regulation, so ciRS-7 may become an important regulatory factor affecting the growth and reproduction of tumor cells. Another miRNA molecule, Sry, which acts as a sponge of circRNA molecules, has similar characteristics and functions (Chen et al., 2021). It contains 16 binding sites of miR-138, which weaken the inhibition of miR-138 by competitive binding to miR-138. Gao et al. (2020) found that circPARD3 triggered tumor progression by inhibiting autophagy in LSCC; CircCORO1C prevented tumor progression via controlling let-7c-5p/PBX3 signal pathway activation, which would be an underlying biomarker in LSCC (Wu et al., 2020). The research on the function and mechanism of circRNA in the pathogenesis of disease is still in its infancy.

In previous research, circRNA_PVT1 (circ_PVT1) was seen as an oncogenic in varieties of cancer, such as breast cancer (Bian, 2019), gastric cancer (Wang et al., 2021), and non-small cell lung cancer (Qin et al., 2019). In the pre-experiment, we found circ_PVT1 was upregulated in LSCC via chip data analysis. We would explore the mechanism of circ_PVT1 on LSCC. Here, we investigate the underlying molecular mechanism in LSCC, which would provide a potential therapy for LSCC.

## Materials and Methods

### Human Sample

Thirty paired tumor tissues and adjacent normal tissues were collected from LC patients. The clinical details of patients are shown in Table 1. Informed consent was obtained from all individuals, and the research protocols obtained approval from the Guangzhou Red Cross Hospital, Jinan University.

**TABLE 1:** Clinical Features of LC Patients.

**Features**	**(N)**	**Percentage**
Age(year)		
<50	18	60%
>50	12	40%
Sex		
Male	22	73.3%
Female	8	26.7%
Lymph node metastasis		
Yes	17	56.7%
TNM stage group		
<lll	20	66.7%
≥lll	10	33.3%
Histology		
Squamous	27	90%
Adenocarcinoma	3	10%
Histological grade		
Wel/moderate	25	83.3%
Poor	5	16.7%

### Cell Culture

Human nasopharyngeal epithelial cells NP69 and LC cells TU-212 and Me-4 were purchased from the typical culture preservation center of NTCC, and LC Hep-2 was purchased from the Cell Resource Center of the Institute of Basic Medicine, Chinese Academy of Medical Sciences. Hep-2 cells were cultured in EMEM medium, TU-212 and Me-4 cells were cultured in RPMI 1640 medium, and NP69 cells were cultured in Defined Keratinoeyte SFM medium. The culture conditions were 37°C and 5% CO_2_. The cells were seeded into 6-well plate according to the amount of 1 × 10^5^/well, and plasmid/NC was transfected into cells. The follow-up experiment was carried out after 48 h of conventional condition culture.

### Western Blot

#### QRT-PCR

The total RNA of cells and tissues extracted by TRIzol was 2 μg total RNA, 4 μL 5× PrimeSeript IV eDNA Synthesis Mix, and 2 μL. Random 6 mers were equipped with 20 μL reverse transcription system (RNase-free dH_2_O complement), and then qPCR reaction system was carried out with DNA template 2 μL. TB Green Fast qPCR Mix 12.5 μL, upstream and downstream primers 1 μL, aseptic H_2_O up to 25 μL reaction conditions: 95°C, 30 s and 40 cycles of 95°C, 5 s and 60°C, 10 s. The relative expression of the target gene was expressed by 2 ^–△^^△^^*Ct*^ method with CAPDH as the internal control.

#### CCK-8

After transfection, the cell suspension with a concentration of 5 × 10^4^/mL was digested with trypsin and inoculated into a 96-well plate with 100 μL per well. After conventional culture for a different periods of time (24, 48, and 72 h), 10 μL CCK-8 solution was added to each well and incubated for 2 h. The absorbance value at 450 nm was measured by enzyme labeling instrument, and the curve was drawn.

#### ELISA

The activity of Caspase-3 was detected by colorimetric protease assay kit (the lysate of CPP32/caspase-3 colorimetric protease assay kit), 1 × 10^6^ cells was added to the luminescent matrix in the equilibrium solution of pNA-Substrate containing 200 μmol/L, and this was then incubated at 37°C for 60 min. The absorbance at the wavelength of 405 nm was measured on the automatic enzyme labeling instrument and analyzed.

### Clone Formation Assay

The cells were re-suspended into cell suspension by trypsin digestion 6 h after transfection and inoculated in a 6-well plate with 3 × 10^3^/well, and 2 mL complete medium was added. The plate was placed into the incubator and allowed to continue to culture. When the number of cells in the cell mass reached 50, the culture was stopped, fixed with methanol for 30 min, stained with 0.1% crystal violet for 20 min, and photo counting was then carried out.

### EdU Staining Assay

The cells were seeded on a 24-well plate covered with slides with a density of 2 × 10^4^/slide with 1 mL medium and climbed overnight. 5, 10, and 25 μmol/L genistein were pretreated with genistein for 24 h, then EdU labeling were performed 3 days later. The fresh medium was mixed with the medium in the culture plate in the same volume, and EdU was added into the incubator to make the final concentration of 10 μ mol/L. The medium in the incubator continued to culture at 37°C for 1 h. 3.7% paraformaldehyde was fixed for 15 min, then PBS was added and washed twice. The 0.5%TritonX-100 membrane was broken for 20 min, then PBS was added and washed twice. Freshly prepared click reaction system (including 1 mol/L pH8.5 Tris-HCl 50 μL, 25 mmol/L CuSO4, 20 μL, 10 mmol/L 6-FAM-Azide, 2.5 μL, 0.5 mol/L ascorbic acid 50 μL, deionized water supplement system to 500 μL) was added. The medium was incubated at room temperature without light for 30 min, then PBS was added and washed three times. 1 μg/mL DAPI was incubated away from light for 10 min, then PBS was added and washed three times. Images were observed and obtained under the microscope. Images were analyzed with Image J software.

### Transwell Assay

Transwell chamber (8.0 μm aperture) was used to evaluate the invasive ability of cells. The transfected cells were re-suspended with serum-free medium after overnight starvation. 100 μL single cell suspension containing 2 × 10^4^ cells was seeded into the upper cavity pre-covered with Matrigel matrix glue, and the lower cavity was added with human fetal bovine serum 600 μL for 48 h. The cells were fixed and stained with crystal violet, and the invading cells were counted randomly in each sample.

### Wound Healing Assay

The transfected cells were seeded into 6-well plate (1 × 10^5^/well). The cells were routinely cultured in the incubator until the cells converged. The monolayer of cells was scratched with 200 μL pipette, and the scratched cells were washed and removed by PBS. After 24 h of culture, regions were randomly selected by inverted microscope to obtain images and measure the width of scratches.

### Flow Cytometry

The transfected cells were collected. Human Annexin V-FITC5 μL was added to the cell suspension; 10 μg/mL propidium iodide (PI) 10 μL was also added, and the mixture was reacted under 4 C light avoidance for15 min, and 4°C mixed buffer 200 μL were added. The cell cycle and apoptosis rate were detected by guava microflow cytometry with Nex-in program.

### Luciferase Assay

The 293T cells were co-transfected with miR-21-5p mimic/miR-NC and circ_PVT1 WT/mutant/CBX4 WT/mutant, and the cells were collected 48 h after transfection. The luciferase activity was determined by promega double luciferase reporter gene kit, and the relative luciferase activity was calculated.

### Xenograft Tumor Model

Hep-2 cells transfected in logarithmic phase were collected, centrifuged, and washed into cell suspension (2 × 10^7^/mL). Three to four week male BALB/e (nu/nu) nude mice were randomly divided into different groups and subcutaneously injected with 0.2 mL of cell suspension on the back of the right hindlimb. The short diameter (A) and long diameter (B) of the tumor were measured with Vernier caliper every 5 days after inoculation. The tumor volume was calculated as BA^2^/2. The mice were observed continuously for 30 days. The animal study was reviewed and approved by Guangzhou Red Cross Hospital, Jinan University.

### Immunohistochemical

Paraffin-embedded slices (about 5 μm thick) were baked at 58°C for 18 h and then pressed with xylene for 15 min. They were then pressed with xylene I 15 min, anhydrous ethanol I for 5 min, anhydrous ethanol II 5 min, 95% ethanol for 5 min, and 85% ethanol for 5 min. Then75% ethanol for 5 min was sequentially dewaxed to water, and after PBS was washed twice; the slices were repaired by microwave in citric acid buffer with PH 6.0. Primary antibody was added to 1: 50, and normal sheep serum and pre-absorbed antibody were used as negative control. Horseradish peroxidase labeled donkey anti-sheep second antibody was incubated in 37°C wet box. DAB was added for 10 min, then dried and sealed with neutral gum. The cases were observed under microscope.

### Statistical Analysis

Prism 8.0 software was used for statistical analysis; the data were expressed as mean ± SEM. *T*-test was used for the comparison between the two groups, and one-way ANOVA was used for the comparison among the groups. *P* < 0.05 was statistically significant, and the experiments were repeated more than 3 times.

## Results

### Circ_PVT1 Is Upregulated in LC Tissues and Cells

**FIGURE 1:**
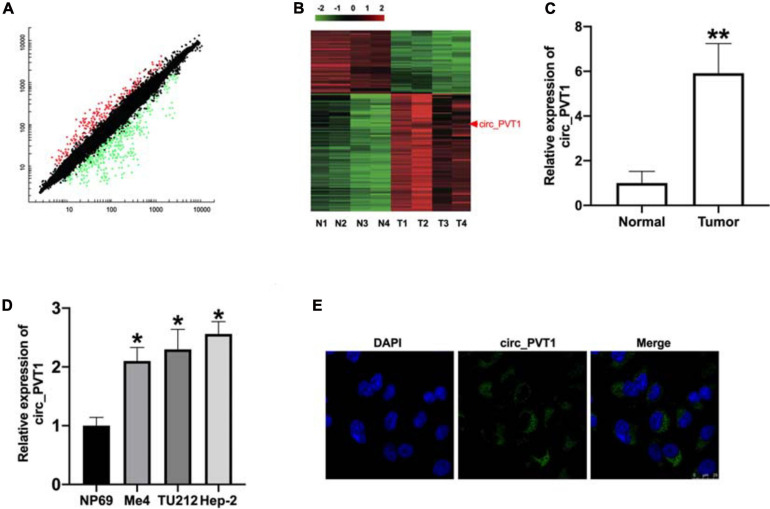
The upregulated level of circ_PVT1 in LC tissues and cells. **(A)** The differentially expressed circRNAs are shown in LC tumor tissues and adjacent normal tissues. **(B)** RNA-seq identified differentially expressed circRNAs’ were cross-matched with a microarray generated from four tumor samples and four normal tissues. **(C)** The expression of circ_PVT1 was detected in LC patients’ tumor tissues and adjacent normal tissues. **P < 0.01, *n* = 30. **(D)** The level of circ_PVT1 in LC cells (Me4, TU212, Hep-2), NP69 cells was indicated as control. *P < 0.05, *n* = 5. **(E)** The subcellular localization of circ_PVT1 in Hep-2 cells.

The paired tumor tissues and adjacent normal tissues were collected from LC patients (Table 2). The QRT-PCR assay performed demonstrated that circ_PVT1 was one of the most differentially expressed in LC tumor tissue compared with adjacent normal tissues (Figures 1A,B). Then we collected 30 paired LC patient tumor tissues and normal tissue; qRT-PCR was employed to assess the expression of circ_PVT1. The result shows that circ_PVT1 was upregulated in LC tumor tissue (Figure 1C). Next, we cultured LC cell lines (Me4, TU212, and Hep-2) and detected the expression level of circ_PVT1. NP69 cells was indicated as control. We found that the expression was increased in Me4, TU212, and Hep-2 cell lines (Figure 1D). Further, we found that circ_PVT1 was localized in cytoplasm (Figure 1E).

**TABLE 2:** Top 10 circRNAs that show differential expression in LC compared with paired normal tissues in patients.

**circRNAs**	**Fold-change**	***P*-value**	**Host gene**
Has_circRNA_0009143	11.1	0.02112	PVT1
Has_circRNA_0000008	9.2	0.03432	GNB1
Has_circRNA_0001412	8.72	0.04728	FIP1L1
Has_circRNA_0001496	7.53	0.01298	CCDC125
Has_ circRNA_ 102049	5.67	0.03789	TADA2A
Has_ circRNA_ 104075	−4.9	0.00765	NUP153
Has_ circRNA_ 102922	−4.3	0.02872	SGPP2
Has_circRNA_0078090	−3.9	0.00182	GRP126
Has_circRNA_0091964	−3.2	0.01273	FLNA
Has_circRNA_0085473	−3.0	0.00023	ATAD2

### Silencing of circ_PVT1 Prevents Proliferation, Migration, and Invasion in LC Cells

**FIGURE 2:**
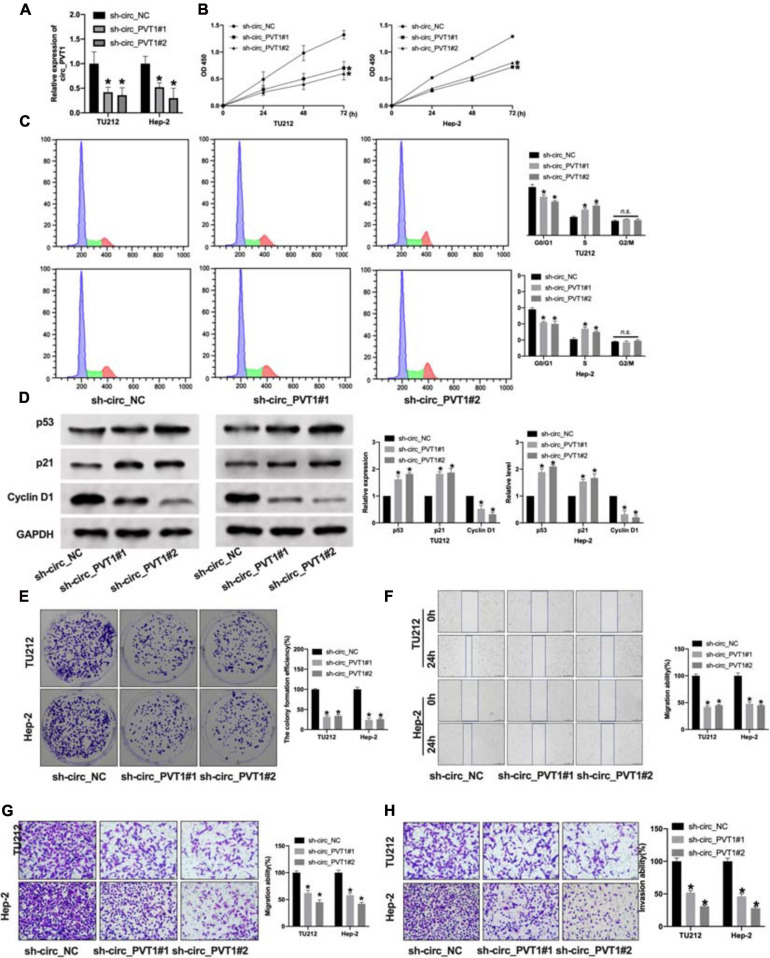
Silencing of circ_PVT1 inhibits proliferation, migration, and invasion in LC cells. **(A)** The knockdown efficiency of shRNA circ_PVT1 (sh-circ_PVT1#1, sh-circ_PVT1#2) was detected by qRT-PCR. *P < 0.05, *n* = 5. **(B)** CCK-8 assay was used to detect the cell viability in TU212 and Hep-2 cells. *P < 0.05, *n* = 5. **(C)** Flow cytometry was performed to assess the cell cycle in TU212 and Hep-2 cells. *P < 0.05, *n* = 5 (*n.s.* as no significant). **(D)** The protein level of p53, p21, and Cyclin D1 in TU212 and Hep-2 cells after sh-circ_PVT1 knockdown. *P < 0.05, *n* = 5. **(E)** The clone formation of LC cells after sh-circ_PVT1#1 and sh-circ_PVT1#2 knockdown. *P < 0.05, *n* = 5. **(F)** Wound healing assay was performed to assess the migration ability. *P < 0.05, *n* = 4. **(G)** Transwell migration assay was used to determine the migration ability. *P < 0.05, *n* = 4. **(H)** Transwell invasion assay was used to determine the invasion ability. *P < 0.05, *n* = 4.

To explore the function of circ_PVT1 in LC progression, we constructed shRNA for inhibiting the expression in LC cells. The knockdown efficiency of shRNA was confirmed by qRT-PCR. Compared with the sh-circ_NC group, sh-circ_PVT1#1 and sh-circ_PVT1#2 could inhibit the expression of circ_PVT1 (Figure 2A). CCK-8 assay revealed that sh-circ_PVT1 inhibited cell viability in TU212 and Hep-2 cells (Figure 2B). Cell cycle assay exposed that silencing of circ_PVT1 prevented cells from transferring from G0/G1 phase into S phase in LC cells (Figure 2C). Then we detected the level of proliferation-associated protein (p53, p21, and Cyclin D1); knockdown of circ_PVT1 inhibited the expression of p53 and p21 and induced the expression of Cyclin D1 in LC cells (Figure 2D). The colony formation assay performed showed that sh-circ_PVT1#1 and sh-circ_PVT1#2 blocked the colony formation in TU212 and Hep-2 cells (Figure 2E). As Figures 2F,G show, sh-circ_PVT1 could prevent migration ability by using wound healing and Transwell assay. Furthermore, Transwell invasion assay described that circ_PVT1 blockage inhibited the invasion ability in LC cells (Figure 2H). In summary, knockdown of circ_PVT1 inhibited the proliferation, migration, and invasion in LC cells.

### Silencing of circ_PVT1 Induces Apoptosis in LC Cells

**FIGURE 3:**
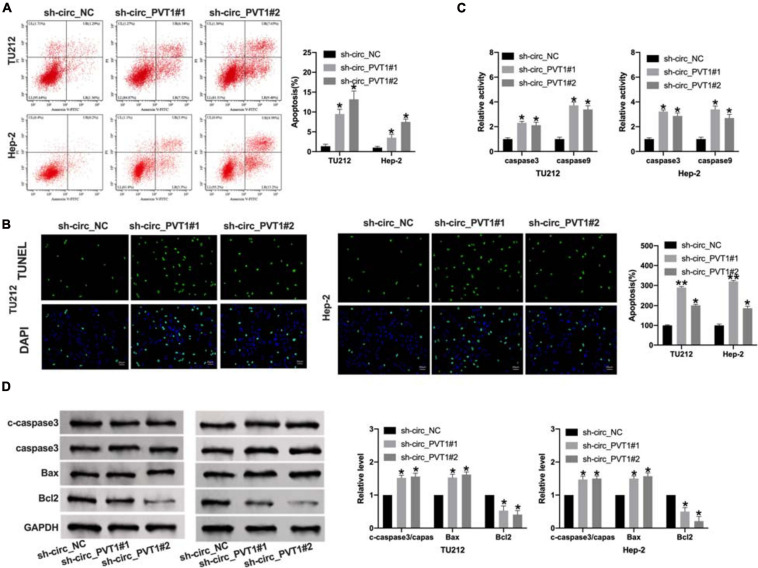
Circ_PVT1 blockage induces apoptosis in LC cells. **(A)** Flow cytometry was performed to detect the apoptosis rate. *P < 0.05, *n* = 4. **(B)** TUNEL staining. *P < 0.05, **P < 0.01, *n* = 4. **(C)** The caspase 3 and caspase 9 activity was determined by ELISA. *P < 0.05, *n* = 4. **(D)** The protein level of cleaved-caspase 3 (c-caspase 3), caspase 3, Bax, and Bcl2 was detected in LC cells after sh-circ_PVT1#1 and sh-circ_PVT1#2 knockdown. *P < 0.05, *n* = 4.

The promotion of tumor cell apoptosis could alleviate the development of tumors. We found that sh-circ_PVT1#1 and sh-circ_PVT1#2 promoted apoptosis positive cells by flow cytometry and TUNEL staining (Figures 3A,B). Then we used ELISA kit to determine the level of caspase 3 and caspase 9. Silencing of circ_PVT1 promoted caspase 3 and caspase 9 in LC cells (Figure 3C). Western blot was carried out to assess the expression level of apoptosis level; the up-regulated cleaved-caspase 3, Bax, and down-regulated Bcl2 was observed in Figure 3D. Taken together, silencing of circ_PVT1 promoted apoptosis in LC cells.

### Circ_PVT1 Regulates wnt4/β-Catenin Signal Pathway in LC Cells

**FIGURE 4:**
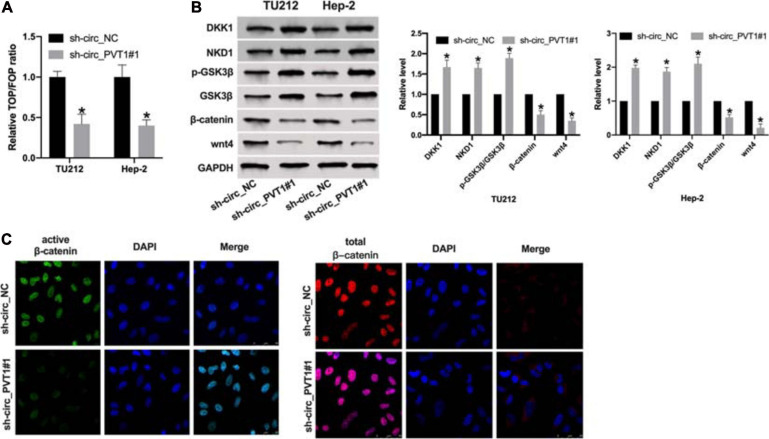
Silencing of circ_PVT1 inhibits wnt/β-catenin signal pathway. **(A)** The LOP/FOP luciferase assay. *P < 0.05, *n* = 4. **(B)** The protein level of DKK1, NKD1, phosphorylation-GSK3β (p-GSK3β), GSK3β, β-catenin, and wnt4 in LC cells. *P < 0.05, *n* = 4. **(C)** Subcellular localization of active β-catenin and total β-catenin in Hep-2 cells by immunofluorescence staining.

Wnt/β-catenin signal pathway plays a key role in tumor progression. By performing TOP/FOP luciferase assay, we found that knockdown circ_PVT1 inhibited transcriptional activity mediated by β-catenin in LC cells (Figure 4A). Then we detected the component levels of wnt/β-catenin signal pathway. The upregulation of DKK1, NKD1, and phosphorylation-GSK3β and the downregulation of β-catenin and wnt4 was observed in sh-circ_PVT1#1 transfected Hep-2 cell (Figure 4B). Therefore, we found that expression of active β-catenin, but not total β-catenin, was reduced in sh-circ_PVT1#1-transfected Hep-2 cells by immunofluorescence (Figure 4C).

### MiR-21-5p/CBX4 Could Be the Downstream of circ_PVT1

**FIGURE 5:**
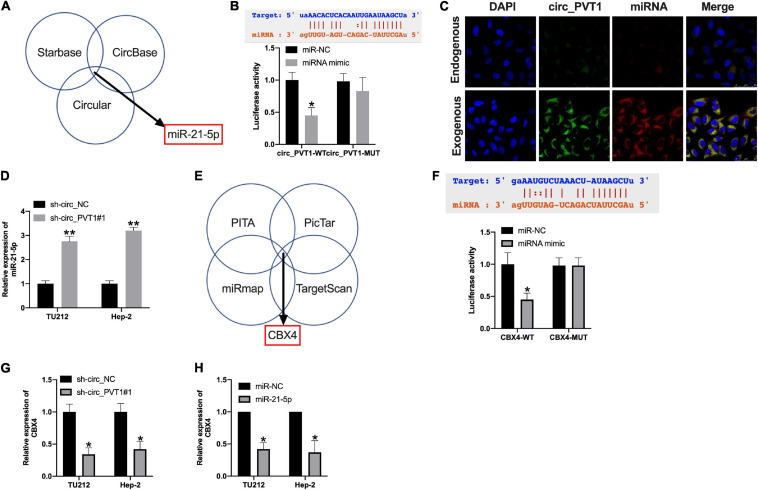
Circ_PVT1 could target miR-21-5p and regulate CBX4. **(A)** Bioinformatics websites (Starbase, CircBase, and Circular) identified miR-21-5p as circ_PVT1 target. **(B)** The predicted binding site between circ_PVT1 and miR-21-5p (upper). The luciferase assay confirmed the relationship between circ_PVT1 and miR-21-5p (lower). ^∗^*P* < 0.05, *n* = 4. **(C)** FISH assay confirmed the co-localization of circ_PVT1 and miR-21-5p. **(D)** The level of miR-21-5p was detected in Hep-2 cells after circ_PVT1 knockdown. ^∗∗^*P* < 0.01, *n* = 4. **(E)** Bioinformatics websites (PITA, PicTar, miRmap, and TargetScan) identified CBX4 as a miR-21-5p target. **(F)** The predicted binding site between CBX4 and miR-21-5p (upper). The luciferase assay confirmed the relationship between CBX4 and miR-21-5p (lower). ^∗^*P* < 0.05, *n* = 4. **(G,H)** The level of CBX4 was explored in TU212 and Hep-2 cells after sh-circ_PVT1 and miR-21-5p transfected. ^∗^*P* < 0.05, *n* = 4.

Bioinformatics website (Starbase, CircBase, and Circular) predicted that miR-21-5p could be an underlying target of circ_PVT1 (Figure 5A). The binding sites are shown in Figure 5B. Luciferase assay revealed that miR-21-5p mimic co-transfected with circ_PVT1 wild type (circ_PVT1-WT), but not circ_PVT1 mutant (circ_PVT1-MUT), revealed the decreased luciferase activity in HEK 293 T cells (Figure 5B). Fish assay performed showed that circ_PVT1 could co-locate with miR-21-5p in Hep-2 cells (Figure 5C). The expression of miR-21-5p was upregulated in sh-circ_PVT1 transfected TU212 and Hep-2 cells (Figure 5D). CBX4 was forecasted as the target of miR-21-5p by bioinformatics website (PITA, PicTar, miRmap, and TargetScan) (Figure 5E). Luciferase assay reported that miR-21-5p could bind with 3’ UTR of CBX4, which indicated the interacting relationship between miR-21-5p and CBX4 (Figure 5F). Silencing of circ_PVT1 and forced expression of miR-21-5p wound prevent the expression of CBX4 (Figures 5G,H).

### Circ_PVT1 Regulates LC Progression via Targeting miR-21-5p/CBX4 Signal Pathway

**FIGURE 6:**
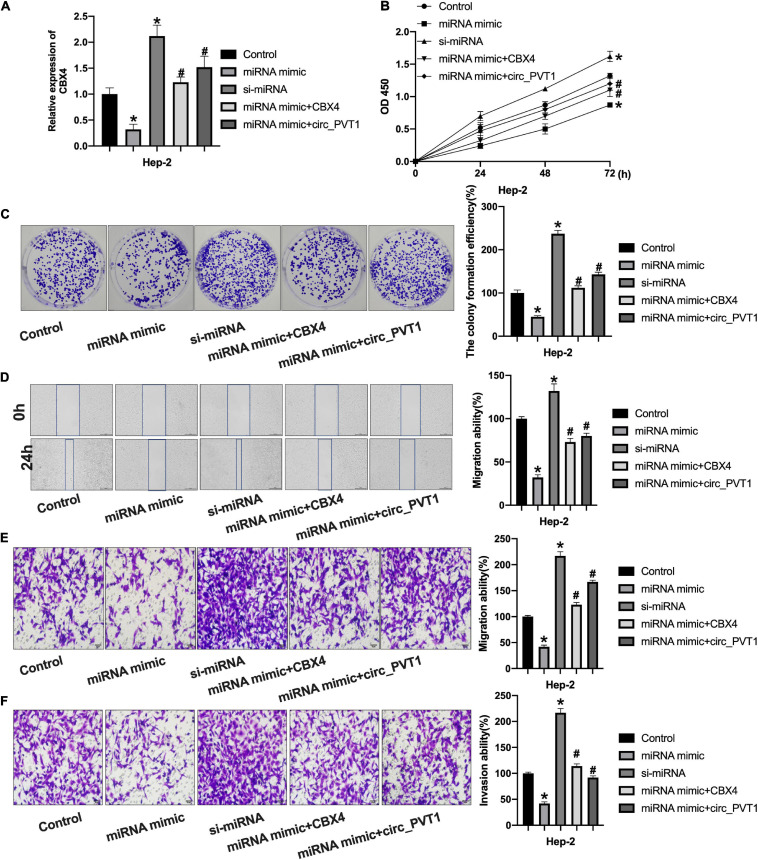
Circ_PVT1 regulated LC cells progression via targeting miR-21-5p/CBX4 signal pathway. **(A)** The level of CBX4 was detected by qRT-PCR. *P < 0.05 vs. Control, ^#^*P* < 0.05 vs. miR-21-5p. *n* = 4. **(B)** The CCK-8 assay was performed in Hep-2 cells. *P < 0.05 vs. Control, ^#^*P* < 0.05 vs. miR-21-5p. *n* = 4. **(C)** The clone formation assay was performed in Hep-2 cells. *P < 0.05 vs. Control, ^#^*P* < 0.05 vs. miR-21-5p. *n* = 4. **(D)** Wound healing assay was performed in Hep-2 cells. *P < 0.05 vs. Control, ^#^*P* < 0.05 vs. miR-21-5p. *n* = 4. **(E,F)** Transwell migration and invasion assay was performed in Hep-2 cells. *P < 0.05 vs. Control, ^#^*P* < 0.05 vs. miR-21-5p. *n* = 4.

Then we co-transfected miR-21-5p with CBX4 and circ_PVT1 into Hep-2 cells. The expression of CBX4 was detected by qRT-PCR (Figure 6A). CCK-8 assay performed showed that forced expression of miR-21-5p would inhibit cell viability which would be prevented by CBX4 and circ_PVT1 (Figure 6B). MiR-21-5p inhibited the clone formation in Hep-2 cells which was abolished by CBX4 and circ_PVT1 (Figure 6C). As Figures 6D,E show, miR-21-5p inhibited the migration ability of Hep-2 cells which was blocked by CBX4 and circ_PVT1. MiR-21-5p inhibited Hep-2 cells invasion which was prevented by CBX4 and circ_PVT1 (Figure 6F). Taken together, circ_PVT1 regulated LC progression via targeting miR-21-5p/CBX4.

### Circ_PVT1 Blockage Prevents Tumor Growth *in vivo*

**FIGURE 7:**
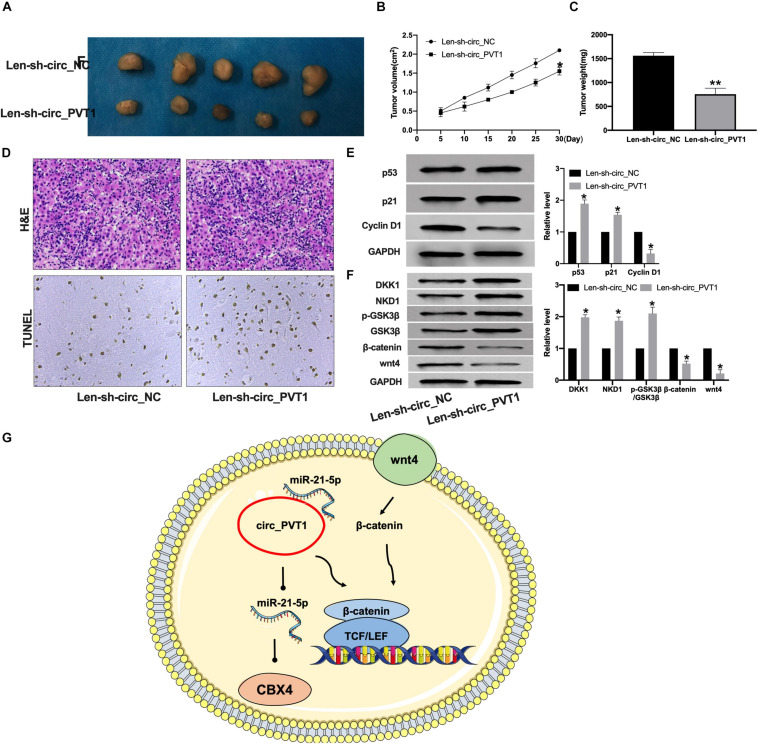
Silencing of circ_PVT1 inhibits tumor growth *in vivo*. **(A)** The tumor image in Len-sh-circ_NC and Len-sh-circ_circ_PVT1. *n* = 5. **(B,C)** The tumor volume and weight were measured in Len-sh-circ_NC and Len-sh-circ_circ_PVT1 group. *P < 0.05. *n* = 5. **(D)** H&E staining in tumor tissues. **(E)** The protein level of p53, p21, and Cyclin D1 was detected in tumor tissues. *P < 0.05, ^∗∗^*P* < 0.01. *n* = 5. **(F)** The protein level of DKK1, NKD1, phosphorylation-GSK3β (p-GSK3β), GSK3β, β-catenin, and wnt4 in tumor tissues. *P < 0.05, *n* = 4. **(G)** Model patterns of circ_PVT1/miR-21-5p/CBX4 axis.

To further confirm the function of circ_PVT1 in LC, we constructed lentivirus package plasmid of circ_NC (Len-sh-circ_NC) and circ_PVT1 (Len-sh-circ_PVT1). The mice were subcutaneously injected with 0.2 mL of Hep-2 cell suspension on the back of the right hindlimb. Lentivirus plasmid was injected into the tail vein. The tumor weight and volume were measured (Figures 7A–C). The tumor section was detected by H&E and TUNEL staining.

Len-sh-circ_PVT1 injected tumor demonstrated increased apoptosis (Figure 7D). Len-sh-circ_PVT1 induced p53 and p21 and inhibited Cyclin D1 expression (Figure 7E). We also detected the protein level of wnt/β-catenin components. Len-sh-circ_PVT1 promotedhe texpression of DKK1, NKD1, and p-GSK3β and inhibited wnt4 and β-catenin in tumor (Figures 7F,G).

## Discussion

Laryngeal cancer is a tumor with a high incidence rate. Distant metastasis is not only a characteristic of the tumor, but also the main factor leading to poor prognosis (Mannelli et al., 2016). In recent years, it has been found that the overall prognosis of LC has not significantly improved. The symptoms of early LC are difficult to identify, which sets up obstacles for early diagnosis and treatment (Warner et al., 2014). Therefore, a better understanding of the molecular mechanism driving LC is helpful to find new therapeutic targets, develop targeted drugs, and improve the prognosis of patients.

The expression of circ_PVT1 is upregulated in different tumors. In the related studies on liver cancer, lung cancer, gastric cancer (Chen et al., 2017), and prostate cancer (Umemori et al., 2020), abnormal circ_PVT1 affects the metabolism, metastasis, and immunity of tumor cells. The change of its expression level is related to the prognosis of tumors and can be used as a marker for early diagnosis. The mechanism may be that circ_PVT1 regulates the occurrence and development of tumor in many stages, such as gene transcription, post-transcription, and translation, and then affects the biological characteristics of tumor cells. Circ_PVT1 was upregulated in cervical cancer, while silencing of circ_PVT1 prevented cervical cancer cells’ progression via targeting epithelial-mesenchymal transition pathway (Wang et al., 2020). Qin et al. (2019) found circ_PVT1 was increased in NSCLC tissues and cells via targeting miR-497 and indirectly regulating Bcl2 expression. The increased expression of circ_PVT1 was found in osteosarcoma; circ_PVT1 would induce metastasis via targeting miR-526b/FOXC2 signal pathway (Qin et al., 2019). Chen et al. uncovered that the upregulated circ_PVT1 could promote tumor growth by sponging miR-125, which would be an underlying mechanism of gastric cancer (Chen et al., 2017).

Here, we found that the expression level of circ_PVT1 in LSCC tissues and cells is significantly higher than that in normal controls. Circ_PVT1 blockage inhibited the proliferation, migration, and invasion ability in LC cells. And silencing of circ_PVT1 induced apoptosis in LC cells. These results revealed that circ_PVT1 acted as an oncogene and regulated tumorigenesis of LC.

Wnt/β-catenin signaling pathway exists widely in living organisms and participates in the process of embryonic development and organogenesis. Wnt/β-catenin signal pathway is related to tumorigenesis (Zhang and Wang, 2020). Wnt/β-catenin signal pathway includes extracellular and intracellular parts, in which Wnt 4 is a key extracellular regulatory protein, and β-catenin plays an important role in intracellular signal transduction (Schaefer and Peifer, 2019). Wnt/β-catenin signaling pathway is overactivated in cervical cancer, tongue squamous cell carcinoma (LSSC), gastric cancer, lung cancer, and other cancers (Nusse and Clevers, 2017). Here, we found that silencing of circ_PVT1 inhibited wnt/β-catenin signaling pathway in LC progression.

Research on the molecular mechanism of LC has been heavily focused on in recent years, as it could provide a new avenue for the treatment of LC However, whether it can really become a therapeutic target for LC remains to be further studied.

## Conclusion

Our study firstly revealed that circ_PVT1 was upregulated in LC, and circ_PVT1 participated in the progression of LC through targeting miR-21-5p and regulating the Wnt/β-catenin signaling pathway. Therefore, circ_PVT1 could be used in a novel therapy for LC.

## Data Availability Statement

The original contributions presented in the study are included in the article/supplementary material, further inquiries can be directed to the corresponding author/s.

## Ethics Statement

The studies involving human participants were reviewed and approved by the Guangzhou Red Cross Hospital, Jinan University. The patients/participants provided their written informed consent to participate in this study. The animal study was reviewed and approved by Guangzhou Red Cross Hospital, Jinan University.

## Author Contributions

FY and YL designed the experiments and prepared the manuscript. M-MA and G-JT performed the experiments. J-LH and Z-RZ wrote the manuscript. All authors contributed to the article and approved the submitted version.

## Conflict of Interest

The authors declare that the research was conducted in the absence of any commercial or financial relationships that could be construed as a potential conflict of interest.
